# Serum leptin concentrations are not related to dietary patterns but are related to sex, age, body mass index, serum triacylglycerol, serum insulin, and plasma glucose in the US population

**DOI:** 10.1186/1743-7075-6-3

**Published:** 2009-01-14

**Authors:** Vijay Ganji, Mohammad R Kafai, Erin McCarthy

**Affiliations:** 1 Division of Nutrition, School of Health Professions, College of Health and Human Sciences, Georgia State University, Atlanta, GA 30302-3995, USA; 2 Department of Mathematics, 1600 Holloway Avenue, San Francisco State University, San Francisco, CA 94132, USA; 3 Mercy Hospital and Medical Center, Chicago, IL 60616, USA

## Abstract

**Background:**

Leptin is known to play a role in food intake regulation. The aim of this study was to investigate the relation between serum leptin concentrations and dietary patterns and demographic, lifestyle, and health factors in the US population.

**Methods:**

Data from the third National Health and Nutrition Examination Survey, 1988–1994 were used to study the association between fasting serum leptin and dietary patterns, sex, race-ethnicity, smoking, age, energy and alcohol intakes, body mass index (BMI), plasma glucose, serum triacylglycerol, and serum insulin in 4009 individuals. Factor analysis was used to derive three principle factors and these were labeled as Vegetable, Fruit, and Lean Meat, Western, and Mixed dietary patterns.

**Results:**

Serum leptin concentrations were significantly higher in Vegetable, Fruit, and Lean Meat (8.5 fg/L) and Mixed patterns (8.0 fg/L) compared to Western pattern (6.29 fg/L) (P < 0.0001). When analysis was adjusted for confounding variables, no significant association was observed between serum leptin and dietary patterns (P = 0.22). Multivariate adjusted serum leptin concentrations were significantly associated with sex (higher in women than in men; β = -1.052; P < 0.0001), age (direct relation, β = 0.006, P < 0.0001), BMI, (direct relation, β = 0.082, P < 0.0001), fasting plasma glucose (inverse relation, β = -0.024, P = 0.0146), serum triacylglycerol (direct relation, β = 0.034, P = 0.0022), and serum insulin (direct relation, β = 0.003, P < 0.0001) but not with race-ethnicity (P = 0.65), smoking (P = 0.20), energy intake (P = 0.42), and alcohol intake (P = 0.73).

**Conclusion:**

In this study, serum leptin was not independently associated with dietary patterns. Sex, age, BMI, serum triacylglycerol, plasma glucose, and serum insulin are independent predictors of serum leptin concentrations.

## Background

Leptin (16 kDa protein), a product of the obesity gene (*Ob/Ob*), has generated interest among researchers to examine its role in obesity. Leptin is synthesized and secreted by adipocytes. Circulating leptin concentrations are related to the body fat mass [1]. Leptin has been known to play a role in regulation of energy expenditure and food intake. When energy intake chronically exceeds energy expenditure, the expanding body fat mass secretes leptin in proportion to energy overload. On the other hand, decrease in circulating leptin activates a response to starvation and indicate inadequate amounts of fat energy stored in the adipose tissue [2]. Thus, diet and dietary factors play a direct or indirect role in modulating circulating leptin concentrations.

Although fat mass is directly related to leptin expression, other factors such as alcohol consumption, cigarette smoking, sex, and race-ethnicity are also associated with serum leptin concentrations [3-7]. A few studies documented the role of diet and nutrients in modulating circulating leptin concentrations [8-12]. Reduced carbohydrate intake rather than reduced fat intake has lowered serum leptin in obese humans [10]. Havel et al [11] reported decreased leptin concentration after ingestion of high fat, low-carbohydrate diet. Others observed no association between macronutrient and serum leptin [13-15].

Several recently published epidemiological studies have characterized individual's diet using factor analysis [16-20]. In factor analysis, foods are separated into food groups based on correlations between foods (factors). Each person receives a score for each derived factor. These factor scores are used to characterize the person's adherence to that pattern. Using factor analysis, Newby et al [16] derived three dominant patterns (fiber rich pattern, protein and alcohol pattern, and sweets pattern) in Baltimore Longitudinal Study on Aging. Kerver et al [17] derived two major dietary patterns, i.e., Western (high intake of foods rich in fat) and American-healthy (high intake of vegetables) using the food intake data from the third National Health and Nutrition Examination, 1988–1994 (NHANES III). Feinman et al [21] using the data from the Active Low-Carber Forum (n = 86,000) reported a low-carbohydrate dietary pattern characterized by high intakes of green and non-starchy vegetables and meat and low intakes of fruits.

To our knowledge, the association between dietary patterns and serum leptin in a representative sample of the US population has never been investigated. The usual approach has been looking at the effect of a single nutrient or food item on leptin. The published results relating leptin to dietary, demographic, and lifestyle factors yielded conflicting results. Considering the role of leptin in obesity, it is important to identify the modifiable factors of circulating leptin concentrations. Additionally, the association between serum leptin and lifestyle factors such as cigarette smoking and alcohol consumption is not well understood. Therefore, the aim of this study was to investigate the relation between serum leptin concentrations and dietary patterns, demographic characteristics, lifestyle factors, energy intake, body mass index (BMI), serum triacylglycerol and insulin, and plasma glucose concentrations.

## Methods

### Survey description

Data used in this study were derived from the public use data files released by the National Technical Information Service, Springfield, VA [22-24]. The NHANES III was conducted by the National Center for Health Statistics over a 6-y period in two phases (1988–1991 and 1991–1994) at 99 locations. A sample representative of the US civilian, non-institutionalized population aged ≥ 2 mo was selected using a complex, stratified, multistage, probability cluster sampling design. Of 39695 persons sampled, 33994 were interviewed in their homes and they completed the Household Adult Questionnaire. Additional health, nutrition, and laboratory data were collected from 30818 participants by health professionals during visits to the mobile examination centers (MEC). Mexican Americans (MA), non-Hispanic blacks (NHB), children aged 2–5 y, and individuals aged ≥ 60 y were oversampled to yield more reliable estimates for these specific groups. Detailed description of the survey design and operation is published elsewhere [25].

### Measurements

The Household Adult File included data on sampled person's sex, age, race-ethnicity, BMI, smoking status, and alcohol consumption. The Hispanic category included MA and persons of Hispanic origin. Whether or not the respondent smoked 100+ cigarettes in their lifetime (yes or no) was also collected. Alcohol intake was determined by summing self-reported consumption of beer, wine, and hard liquor. One serving of alcohol was equivalent to 12 ounces (360 mL) of beer, 4 ounces (120 mL) of wine, or 1 ounce (30 mL) of hard liquor.

Blood was collected by venipuncture from participants at the MEC after overnight fasting for varied lengths of time. Specimens were shipped to the Centers for Disease Control and Prevention laboratories for priority analysis. After priority analysis, surplus sera were frozen at -70°C. The sera went through at least 1 freeze-thaw cycle during an average of 8 y of storage before leptin concentrations were measured. Leptin was previously shown to remain stable through 5 freeze-thaw cycles and even after storage for as long as 29 y [26]. Leptin concentrations were only measured in the surplus sera for persons who had fasted ≥ 9 h and age > 20 y. Serum leptin was analyzed by Linco Research, Inc., St. Louis, Missouri [27]. The minimum detectable concentration of the leptin assay was 0.5 fg/L and the limit of linearity was 100 fg/L. Coefficients of variation for within- and between assays ranged from 3.4% to 8.3% and from 3.6% to 6.2%, respectively. Serum insulin was measured with radioimmunoassay method, plasma glucose was measured spectrophotometrically (glucose hexokinase method), and serum triaclyglycerol was measured with microbial lipase method [28].

### Dietary assessment

In NHANES III, dietary intake data were collected using an 80-item qualitative food frequency questionnaire (FFQ). Depending on the respondent's preferences, FFQ was administered either in English or Spanish by trained interviewers. The FFQ was pre-tested and modified to be culturally appropriate for non-Hispanic whites (NHW), NHB, and MA. Participants were asked the average number of times foods consumed during the 1-mo period preceding the respondent's interview date. Data on frequency of consumption were collected for milk and milk products, meat, eggs, fruits and juices, vegetables, grains and cereals, legumes, desserts and sweets, beverages, and fats. Participants were excluded whose interviews were coded as unreliable and participants who did not answer the FFQ. For the purpose of this study, foods from the FFQ were categorized into 25 food groups as described in Table 1. Frequency of intake of these 25 food groups were used to determine dietary patterns.

**Table 1:** Food groups used in the dietary pattern analysis: the NHANES III ^1^

**Food Groups^2^**	**Foods from the Food Frequency Questionnaire^3^**
Low-fat dairy	1% milk, 2% milk, skim milk, and yogurt/frozen; Swiss, cheddar, and cottage cheeses
High-fat dairy	Chocolate milk, whole milk, ice cream, ice milk, milk shakes, and cheese dishes
Pizza/lasagna	Pizza, calzone, and lasagna
Soups	Stew or soup with vegetables
Processed meats	Bacon, sausage, lunch meats, liver, and organ meats
Meats	Beef, hamburger, steaks, pork, and ham
Fish and other sea food	Shrimp, clams, oysters, and lobster; Fish: fillets, sticks, and tuna
Poultry	Chicken (all types)
Egg	Scrambled, fried, and omelettes
Fruit/fruit juices	Fruits: oranges, grape fruit, cantaloupe, honey dew, water melon, peaches, nectarines, apricots, mango, apples, bananas, pears, berries, grapes, and strawberries; Juices: Orange, grapefruit, tangerine, grape, apple, and cranberry
Starchy vegetables	White potatoes, French fries, potato salad, sweet potatoes, yams, squash, and carrots
Tomatoes	Tomatoes including fresh and stewed tomatoes, tomato juice, and salsa
Cruciferous and green vegetables	Broccoli, Brussel sprouts, cauliflower, cabbage, coleslaw, sauerkraut, spinach, greens, kale, and tossed salad
Other vegetables	Hot red chili peppers, peppers (green, red, and yellow), green beans, corn, peas, mushrooms, and zucchini
Legumes	Beans, lentils, kidney, pinto, refried, black, and baked
Nuts	Peanuts, peanut butter, nuts, and seeds
Cereal	Cooked, hot cereals, oatmeal, cream of wheat/rice, grits, and cold breakfast cereals
Whole grains	Dark breads, whole wheat, and rye
Refined grains	White bread, rolls, English muffins, crackers, corn bread, corn muffins, corn/flour tortillas, and rice
Snacks and sweets	Salted snacks, potato chips, pretzels, popcorn, cakes, cookies, brownies, pies, doughnuts, chocolate candies, and fudge
Fats	Oil and vinegar, mayonnaise, and salad dressings
High energy drinks	Hi-C^®^, Tang^®^, Hawaiian Punch^®^, regular colas, and sodas
Low-energy drinks	Diet colas/sodas and crystal light
Coffee/tea	Coffee and tea
Alcohol	Beer, wine, and hard liquor

^1 ^n = 4009; weighted n = 99,249,927

^2 ^Foods consumed by survey participants were categorized into 25 food groups.

^3 ^Food intake data were collected by using an 80-item qualitative Food Frequency Questionnaire.

Additionally, one 24-h dietary recalls were collected using an automated, microcomputer-based dietary interview and coding system. Participants reported all foods and beverages consumed except plain water for the previous 24-h time period. Nutrient composition of foods reported in food recalls was based on the USDA Survey Nutrient Databases [29]. A number of quality control measures were employed to ensure the accuracy of food recalls. [30]. In this study, energy intake from the food recalls was used as a potential determinant of serum leptin.

### Study sample

The current study sample initially consisted of 6415 participants derived from persons whose fasting serum leptin concentrations were measured in surplus sera. Pregnant (n = 288) and lactating (n = 95) women and diabetic persons (n = 1498) and individuals identified as other race-ethnicity were excluded from the analysis (n = 187). Persons who responded 'yes' to a question whether they were diabetic or whether they were taking diabetic medication were treated as persons with diabetes. Individuals with missing values for sex, race-ethnicity, age, smoking, alcohol and energy intakes, BMI, serum triacylglycerol, plasma glucose, serum insulin, and FFQ were also excluded (n = 338). After applying the above criteria, the final study sample consisted of 4009 participants (men, 1907; women, 2102; NHW, 1907; NHB, 1076; MA, 1026). The average age of the participants was 43.4 ± 0.89 (mean ± SE)

### Data analysis

Statistical analyses were performed with SUDAAN (SUDAAN for Windows, version 8.0.2, Research Triangle Institute, Research Triangle Park, NC) and SAS software (Statistical Analysis Software for Windows, version 9.1, Cary, NC) packages. Use of SUDAAN had allowed us to account for the complex survey design. Sample weights were used to account for unequal probabilities of selection, cluster design, and planned oversampling of selected subgroups. Detailed guidelines on the use of sample weights in data analysis are described in NHANES III Analytical and Reporting Guidelines [25].

**Figure 1:**
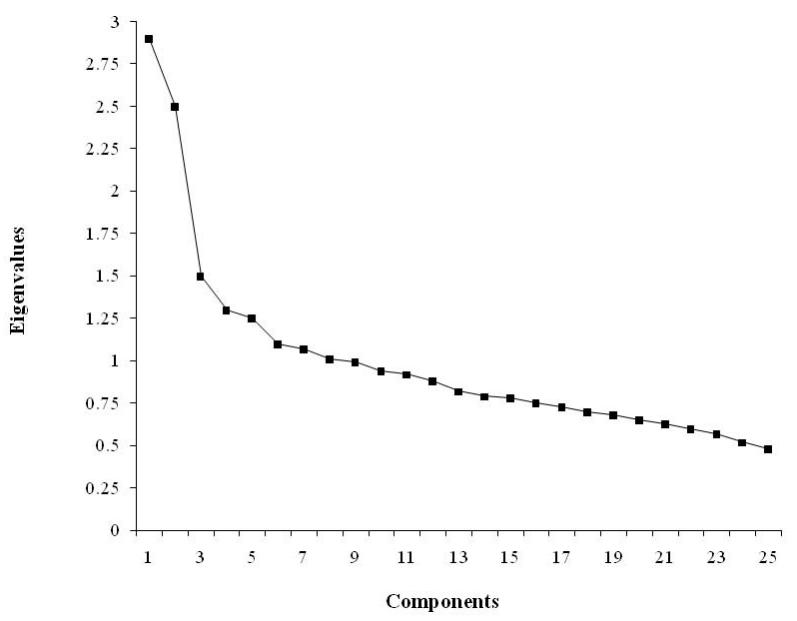
**Catell Scree Plot **Based on the frequency of intake of 25 food groups from the NHANES III (n = 4009; weighted n = 99,249,927). Factor components are on the X axis and the corresponding eigenvalues are on the Y axis. Scree plot was used to determine the number of factors to be extracted. Only first three factors (patterns) were retained in the analysis because the plot starts flattening after the first 3 factors.

**Figure 2:**
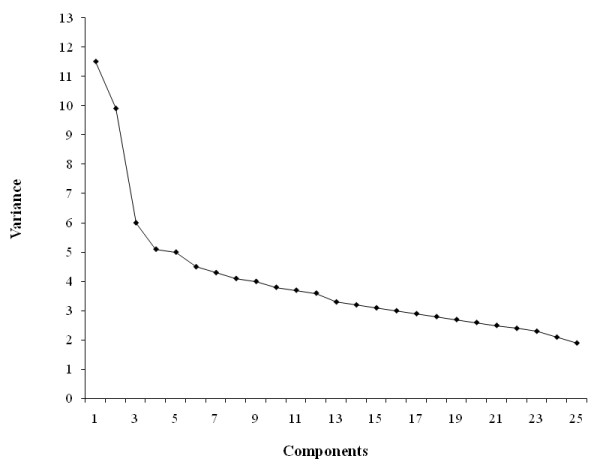
**Factor (Principle component) analysis **(n = 4009; weighted n = 99,249,927). Factor components are on the X axis and the corresponding variances are on the Y axis. Eigenvalues ≥ 1.5 and explained variance ≥ 6% were used in determining number factors to be retained. Factor, 1, 2, and 3 explained ≈11.5%, ≈10%, and ≈6%, respectively, variance in food intake.

In SAS, we used the PROC FACTOR procedure for analysis which utilized principle component analysis and varimax rotation, an orthogonal rotation to derive uncorrelated factors. Varimax rotation was used to maximize the variance of the factors. Principle component analysis requires preselection of number of factors to be retained [16,17]. Factor solution was applied to frequency of food intake of the 25 food groups. This yielded eigenvalues for 25 components. Eigenvalues for 25 components/factors are presented in Figure 1. Eigenvalues measure the amount of variance in relation to total variance. Variances for 25 components/factors are presented in Figure 2. We used eigenvalues (≥ 1.5), explained variance (≥ 6%), and Cattell scree plot to determine the number of factors to be extracted. Based on this, we extracted first 3 consecutive factors as these three factors accounted for the most variability. Factor loadings were determined for each food group across 3 factors. A factor score was determined for each of the 3 derived factors based on the food intake frequency of the 25 food groups. Thus, the principle component analysis yielded a factor score for each subject [17]. Factors were named according to the food groups that loaded most positively on the factor [16].

Analysis of variance (ANOVA) for continuous variables and chi-square tests for categorical variables were used to determine if there were differences for any demographic characteristics, lifestyle factors, energy intake, BMI, serum triacylglycerol and insulin, and plasma glucose among the 3 dietary pattern groups. Univariate ANOVA was used to determine if mean serum leptin concentrations differed across dietary patterns for all subjects, men, and women (unadjusted analysis). Analysis of covariance (ANCOVA) was used to determine if serum leptin concentrations differed across the different dietary patterns after adjusting for sex, age, race-ethnicity, smoking, alcohol and energy intakes, BMI, serum triacylglycerol and insulin, and plasma glucose. Multiple comparisons among dietary patterns for serum leptin were made using t-test with a Bonferroni correction (P < 0.0167).

Association between serum leptin concentrations and dietary patterns, demographic variables, lifestyle factors, energy intake, BMI, serum triacylglycerol and insulin, and plasma glucose was determined using multivariate regression analysis. Because serum leptin concentrations were not normally distributed, a logarithmic transformation was used to satisfy the distribution requirements in all analyses. An α of 0.05 was used to designate statistical significance.

## Results

**Figure 3:**
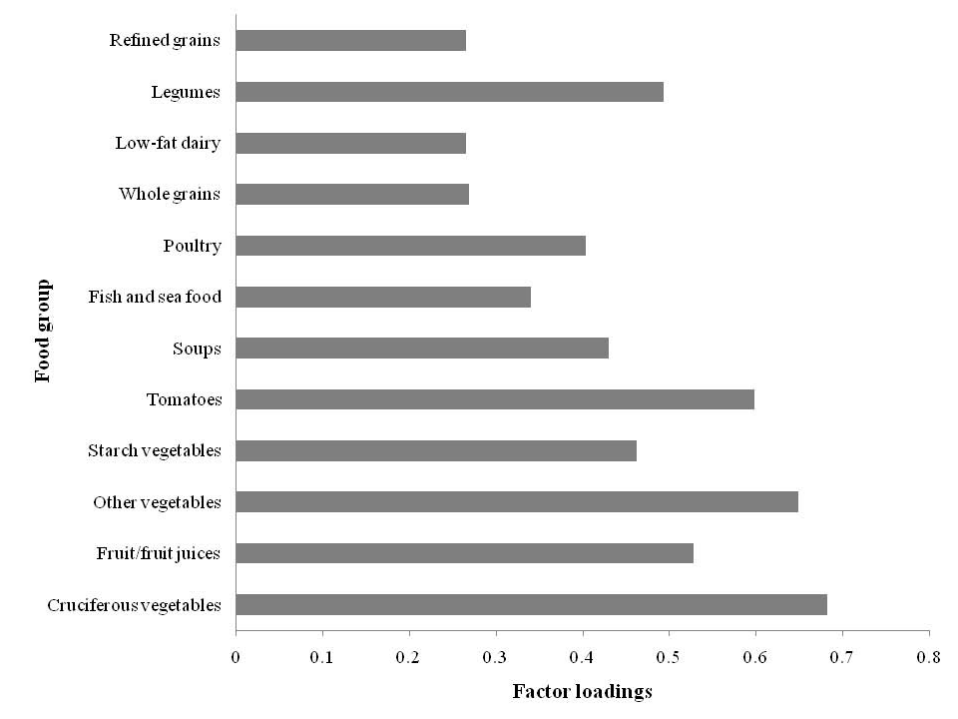
**Factor loadings (correlation coefficients) for Vegetable, Fruit, and Lean Meat dietary pattern in the principle component analysis **(n = 4009; weighted n = 99,249,927). Factor loadings were shown for selected food groups (≥ ± 0.2) for simplicity. Vegetable, Fruit, and Lean Meat (Factor 1) pattern was loaded heavily on vegetables in general, fruits/fruit juices, fish/sea food, poultry, soups, low-fat dairy, and legumes.

**Figure 4:**
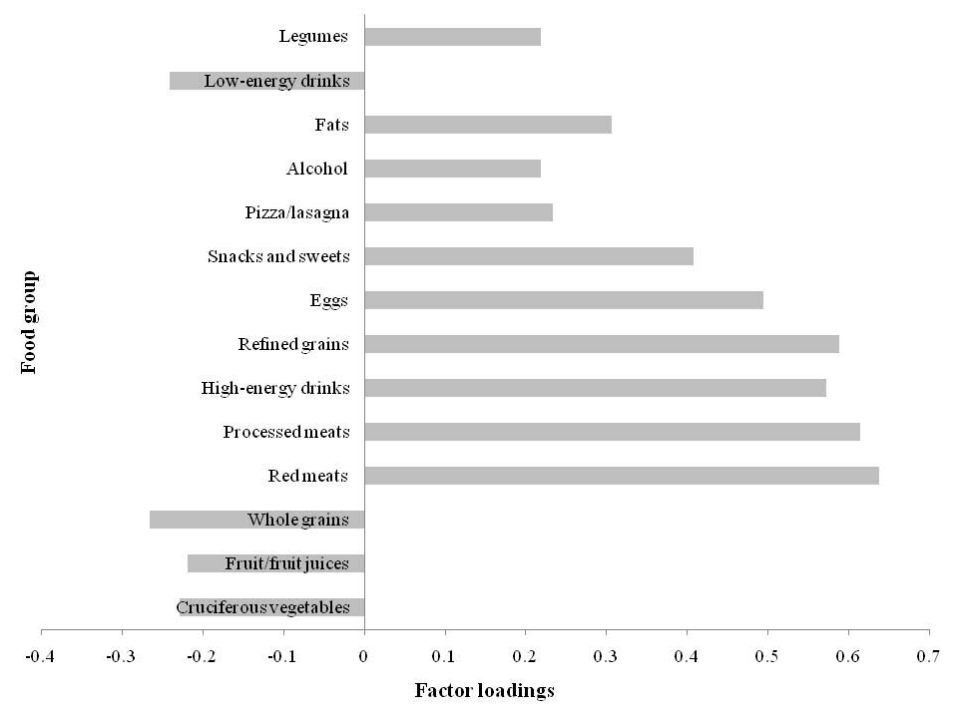
**Factor loadings (correlation coefficients) for Western dietary pattern in the principle component analysis **(n = 4009; weighted n = 99,249,927). Factor loadings were shown for selected food groups (≥ ± 0.2) for simplicity. Western pattern (Factor 2) was loaded heavily on red and processed meats, high-energy drinks, refined grains, pizza/lasagna, eggs, fats, and snacks/sweets.

**Figure 5:**
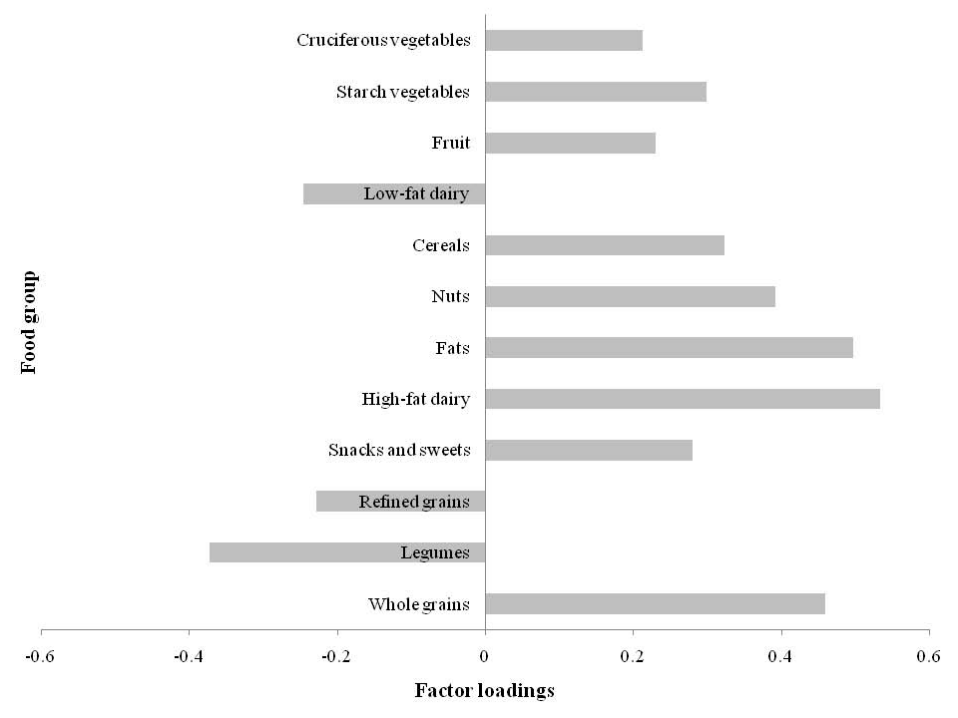
**Factor loadings (correlation coefficients) for Mixed dietary pattern in the principle component analysis **(n = 4009; weighted n = 99,249,927). Factor loadings were shown for selected food groups (≥ ± 0.2) for simplicity. Mixed pattern (Factor 3) was loaded heavily on high-fat dairy, fats, nuts, cereals, starch vegetables, snacks and sweets, and whole grains.

Factor loadings for the 3 factors (patterns) derived are shown in Figures 3, 4, and 5. Higher positive factor loading values contribute most to the factor score and suggest stronger adherence to that pattern. Higher negative values contribute least to the factor score and suggest weaker adherence to that pattern [16]. Factor 1 had higher loadings for vegetables in general, fruits/fruit juices, soups, fish, poultry, whole grains, low-fat dairy, and legumes foods (Figure 3). Factor 2 had higher loadings for red and processed meats, high-energy drinks, refined grains, eggs, snacks/sweets, pizza/lasagna, and alcohol and had negative loadings for cruciferous vegetables, fruits/juices, whole grains, and low-energy drinks (Figure 4). Factor 3 had higher loadings for high-fat dairy, fats, nuts, and cereals (Figure 5). Based on this data, Factor 1, 2, and 3 were named as Vegetable, Fruit, and Lean Meat, Western, and Mixed dietary patterns, respectively. The Vegetable, Fruit, and Lean Meat pattern was the most dominant pattern and explained ≈11.5% of the variance in food intake. Western and Mixed patterns explained ≈10% and ≈6% of the variance of food intake, respectively.

Subjects were categorized into one of 3 dietary pattern groups based on their factor scores. Persons who received the highest factor scores for food groups that constitute Vegetable, Fruit, and Lean Meat pattern (cruciferous, starch, and other vegetables, fruit/fruit juices, tomatoes, soups, fish/seafood, poultry, low-fat dairy, and legumes) were placed in that pattern. Persons who received the highest factor scores for red meat, processed meat, high-energy drinks, refined grains, eggs, snacks/sweets, pizza/lasagna, alcohol, and coffee/tea groups were placed in the Western dietary pattern group. Lastly, persons who received the highest factor scores for high-fat dairy, fats, nuts, whole grains, starch vegetables, snacks and sweets, and cereals groups were placed in the Mixed pattern group.

Characteristics of subjects by dietary patterns are presented in Table 2. Overall, the study sample consisted of 48% men and 52% women. Of 4009 subjects, 47% were NHW, 27% were NHB, and 26% were MA. Vegetable, Fruit, and Lean Meat group consisted of mostly women (58%) and they smoked fewer cigarettes in their lifetime compared to those in Western and Mixed pattern groups. Persons in the Vegetable, Fruit, and Lean Meat group were significantly older (46.2 y) than those in the Western dietary group (38.6 y) (P < 0.0001). Vegetable, Fruit, and Lean Meat group consumed the least amount of alcohol (7.8 drinks/mo). Persons consuming a Western pattern diet were mainly men (56%), were of NHW origin (41%), and smoked slightly more cigarettes in their lifetime (56%) than the other two groups. Western dietary group consumed more alcohol (11 drinks/mo) compared to the Vegetable, Fruit, and Lean Meat group. Western dietary group consumed the greatest amount of energy, while Vegetable, Fruit, and Lean Meat group consumed the least amount of energy. There was no significant difference in BMI, serum triacylglycerol, plasma glucose, and serum insulin across 3 dietary pattern groups.

**Table 2:** Characteristics of study population by dietary patterns in the NHANES III ^1^

	Dietary Pattern			
		
**Characteristic**	**Vegetable, Fruit, and Lean Meat** **(n = 1482)**	**Western** **(n = 1503)**	**Mixed** **(n = 1024)**	P value ^2^
Sex (n, %)				< 0.0001
Men	617 (41.6)	835 (55.6)	455 (44.4)	
Women	865 (58.4)	668 (44.4)	569 (55.6)	
Race-ethnicity (n, %)				< 0.0001
Non-Hispanic white	583 (39.3)	616 (41.0)	708 (69.1)	
Non-Hispanic black	303 (20.5)	521 (34.7)	252 (24.6)	
Mexican American	596 (40.2)	366 (24.3)	64 (6.3)	
Cigarettes smoked (n, %) ^3^				< 0.0001
Yes	635 (42.8)	836 (55.6)	509 (49.7)	
No	847 (57.2)	667 (44.4)	515 (50.3)	
Age (y) ^4^	46.2 ± 1.1 ^5^	38.6 ± 0.7	46.6 ± 0.9 ^5^	< 0.0001
Alcohol intake (drinks/mo) ^4,6^	7.8 ± 0.5	10.8 ± 1.3	8.0 ± 0.7	0.09
Energy intake (kcal) ^4^	2022 ± 40 ^5,7^	2553 ± 57 ^7^	2253 ± 45 ^5^	< 0.0001
Body mass index (kg/m^2^) ^4^	26.4 ± 0.2	26.5 ± 0.3	26.5 ± 0.2	0.98
Serum triacylglycerol (mmol/L) ^4^	1.49 ± 0.06	1.51 ± 0.04	1.49 ± 0.04	0.83
Plasma glucose (mmol/L) ^4^	5.42 ± 0.04	5.32 ± 0.03	5.37 ± 0.03	0.11
Serum insulin (pmol/L) ^4^	61.7 ± 2.5	63.7 ± 2.5	58.8 ± 1.8	0.11

^1 ^n = 4009; weighted n = 99,249,927.

^2 ^Chi-square test was used for categorical variables (sex, race-ethnicity, and cigarettes smoked) and analysis of variance was used for continuous variables (age, alcohol intake, energy intake, body mass index, serum triacylglycerol, plasma glucose, and serum insulin).

^3 ^Persons who smoked 100+ cigarettes prior to the survey.

^4 ^Geometric mean ± standard error.

^5 ^Significantly different from Western dietary group. Bonferroni correction, *P *< 0.0167.

^6 ^One drink of alcohol is defined as 360 mL of beer, 120 mL of wine, or 30 mL of hard liquor. Total alcohol intake was computed by summing self-reported consumption of number of drinks of beer, wine, and hard liquor.

^7 ^Significantly different from Mixed dietary group. Bonferroni correction, *P *< 0.0167.

Serum leptin concentrations according to dietary patterns are presented in Table 3. The unadjusted serum leptin concentrations differed significantly across the three dietary pattern groups for all subjects (P < 0.0001) but did not differ in a separate analysis for men (P = 0.09) and women (P = 0.15). Serum leptin concentrations for all subjects were significantly lower in persons who consumed Western pattern diet compared to those who consumed the Vegetable, Fruit, and Lean Meat and Mixed pattern diets (P = 0.0001). When analysis was adjusted for sex, race-ethnicity, smoking, age, energy intake, alcohol intake, BMI, serum triacylglycerol, plasma glucose, and serum insulin, the association between serum leptin and dietary patterns was no longer significant for all subjects (P = 0.22), for men (P = 0.74), and for women (P = 0.25).

**Table 3:** Serum leptin concentrations by dietary patterns in the NHANES III ^1^

	Dietary Pattern ^2^			
		
	**Vegetable, Fruit and Lean Meat** **(n = 1482)**	**Western** **(n = 1503)**	**Mixed** **(n = 1024)**	P value ^3^
	fg/L	fg/L	fg/L	
Unadjusted analysis				
All subjects	8.50 ± 1.04 ^4^	6.29 ± 1.04 ^5^	8.00 ± 1.04 ^4^	< 0.0001
Men	4.90 ± 1.04	4.26 ± 1.04	4.57 ± 1.04	0.09
Women	11.82 ± 1.04	12.94 ± 1.05	13.07 ± 1.05	0.15
Adjusted analysis				
All subjects ^6^	7.39 ± 1.02	7.69 ± 1.02	7.69 ± 1.02	0.22
Men ^7^	4.44 ± 1.04	4.57 ± 1.03	4.48 ± 1.03	0.74
Women ^7^	12.18 ± 1.03	12.68 ± 1.03	12.81 ± 1.03	0.25

^1 ^n = 4009; weighted n = 99,249,927.

^2 ^Mean ± standard error. Analysis was performed on log leptin concentrations because of non-normality distribution.

^3 ^Significance for the Wald F statistic in the analysis of covariance.

^4 ^Signifcantly different from Western dietary pattern, Bonferroni correction, P < 0.0167.

^5 ^Signficantly different from Mixed dietary pattern, Bonferroni correction, P < 0.0167.

^6 ^Analysis was adjusted for sex, age, race-ethnicity, smoking, alcohol and energy intakes, BMI, serum triacylglycerol and insulin, and plasma glucose.

^7 ^Analysis was adjusted for age, race-ethnicity, smoking, alcohol and energy intakes, BMI, serum triacylglycerol and insulin, and plasma glucose.

Multivariate regression analysis showing the association between serum leptin concentrations and dietary patterns and demographic, lifestyle, and health factors is presented in Table 4. Multivariate adjusted serum leptin concentrations were significantly related to sex (higher in women than in men; β = -1.052; P < 0.0001), age (a positive relation, β = 0.006, P < 0.0001), BMI, (a positive relation, β = 0.082, P < 0.0001), fasting plasma glucose (an inverse relation, β = -0.024, P = 0.0146), serum triacylglycerol (a positive relation, β = 0.034, P = 0.0022), and serum insulin (a positive relation, β = 0.003, P < 0.0001). However, there was no significant association between adjusted serum leptin concentrations and race-ethnicity (P = 0.65), smoking (P = 0.2), energy intake (P = 0.42), and alcohol intake (P = 0.73).

**Table 4:** Association between serum leptin concentrations and dietary patterns, demographic characteristics, and lifestyle and health factors in the NHANES III ^1^

**Variable**	**β^2^**	**SE**	P value ^3^
Dietary Patterns			0.22
Vegetable, Fruit, Lean Meat	-0.042	0.02	
Western	-0.008	0.03	
Mixed (referent category)	--	--	
Sex			< 0.0001
Men	-1.052	0.03	
Women (referent category)	--		
Race-ethnicity			0.66
Non-Hispanic white	0.01	0.03	
Non-Hispanic black	-0.017	0.03	
Mexican American (referent category)	--	--	
Cigarettes smoked ^4^			0.20
Yes	-0.035	0.03	
No (referent category)	--	--	
Age (y)	0.006	0.001	< 0.0001
Energy intake (kcal)	< -0.0001	< 0.001	0.42
Alcohol intake (drinks/mo) ^5^	-0.0002	0.001	0.73
Body mass index (kg/m^2^)	0.082	0.001	< 0.0001
Serum triacylglycerol (mmol/L)	0.034	0.01	0.0022
Plasma glucose (mmol/L)	-0.024	0.01	0.0146
Serum insulin (pmol/L)	0.003	0.001	< 0.0001

^1 ^n = 4009; weighted n = 99,249,927; Analysis was performed on log leptin concentrations because of non-normality distribution.

^2 ^Log regression coefficients. Indicates the magnitude of relationship between serum leptin concentrations and dietary patterns, demographic characteristics, or lifestyle and health factors. A positive coefficient indicates direct relationship and a negative coefficient indicates inverse relationship between serum leptin and aforementioned variables.

^3 ^Significance in the analysis of covariance. Dietary patterns, sex, race-ethnicity, and smoking were used as categorical variables; age, alcohol and energy intakes, BMI, serum triacylglycerol, plasma glucose, and serum insulin were used as continuous variables. Multivariate analysis was adjusted for these variables simultaneously.

^4 ^Persons who smoked 100+ cigarettes prior to the survey.

^5 ^One drink of alcohol is defined as 360 mL of beer, 120 mL of wine, or 30 mL of hard liquor. Total alcohol intake was computed by summing self-reported consumption of number of drinks of beer, wine, and hard liquor.

## Discussion

Using a subset of a nationally sampled survey, we derived 3 dietary patterns, Vegetable, Fruit, and Lean Meat, Western, and Mixed. Dietary patterns derived from this study are remarkably similar to the patterns identified in other studies using factor analysis [16,17,19,20,31]. First, Hu et al [20] and later, Fung et al [19], in the Health Professionals Follow-up Study derived 2 major food patterns, Prudent (high intakes of vegetables, fruit, legumes, whole grains, and fish and other seafood) and Western (high intakes of processed and red meat, butter, high-fat dairy products, eggs, and refined grains). Khani et al [31] identified 3 dietary patterns and labeled them as Healthy (vegetables, fish, poultry, tomatoes, whole-grains, low-fat dairy, etc), Western (processed meats, refined grains, high-fat diary, sweets etc), and Drinker. Using the NHANES III data, Kerver et al [17] derived 2 major food patterns ("American-Healthy" and Western) and 4 minor dietary patterns. They used 35 food groups and had a larger sample size with differing demographic characteristics. Therefore, different dietary patterns emerged in our study. Although there is a slight variation regarding number of factors (patterns) derived from study to study, the first two major dietary patterns, i.e., Prudent (healthy) and Western (not so healthy) have remained by and large consistent over time and between studies.

To our knowledge, this is the first study to examine the relation between dietary patterns and serum leptin in a nationally representative US cohort. Through multivariate-adjusted analysis, we found, dietary patterns are not independently related to serum leptin. In contrast, Fung et al [19] observed a significant positive correlation between the Western pattern and serum leptin (r = 0.28; P < 0.0001) but not between the Prudent pattern and serum leptin (n = 466). Jensen et al [8] reported that persons in the highest quintile for whole-grain intake had 11% lower circulating leptin compared to those in the lowest quintile. Winnicki et al [9] reported that a diet high in fish was associated with lower plasma leptin, independent of body fat or BMI (n = 622). Chu et al [7] observed no association between serum leptin and fat intake after adjustment for energy and BMI. This lends support to the notion that the relation between serum leptin and dietary patterns is confounded by the amount of energy consumed and body weight. Inconsistent results between these studies may partly be explained by differences in subject characteristics, dietary intake assessment methods employed, and confounding variables used in the data analysis.

Although the association between dietary patterns and circulating leptin is unclear, association between macronutrients and serum leptin has been reported. Dietary carbohydrate appears to play a role in leptin metabolism [4,10]. Over a 24-h period, a significant decrease in postprandial leptin concentrations was observed after consuming a high-fat, low-carbohydrate diet [4]. Volek et al [32] reported a 42% decrease in leptin with carbohydrate restricted diet (percentage of energy from carbohydrate:fat:protein = 12:59:28) and a 18% decrease in leptin with low-fat diet (percentage of energy from carbohydrate:fat:protein = 56:24:20) for 12 weeks in overweight subjects. This significant decrease in leptin persisted after normalization of values to body mass and fat mass suggesting that dietary carbohydrate restriction improves leptin resistance more likely due to reduction in body mass [32,33]. Also, carbohydrate restriction lowered concentrations of serum insulin and triacylglycerol even in the absence of weight loss [34]. Thus, the dietary factor/component (for example carbohydrate restriction) that lowers serum leptin can also improve several indicators of metabolic syndrome such as circulating triacylglycerol and insulin and fat mass [32,34]. However, in this current study, the adjusted serum leptin concentrations are not related to dietary patterns.

We found a positive relation between serum leptin and insulin concentrations (P < 0.0001). In an experimental study, leptin concentrations increased after 4–6 h of insulin administration [35]. Changes in circulating leptin concentration in response to fasting, refeeding, and macronutrient intake are likely mediated by insulin-stimulated glucose metabolism in adipose tissue [36]. Expression of leptin from adipocytes is directly related to the glucose uptake by adipocytes. Additionally, inhibition of glucose transport and metabolism in adipocytes markedly decreased *ob *gene expression leading to the reduced production of leptin [36].

Not only that multivariate-adjusted serum leptin was significantly associated with sex but also that sex was the strongest predictor of serum leptin after including the BMI in the model which suggests that metabolic or physiologic differences might contribute to the differences in serum leptin between men and women. Our finding of no association between serum leptin and race-ethnicity is in agreement with some studies [3,37,38]. In contrast, Ruhl et al [39] reported a higher serum leptin in blacks than in whites independent of anthropometric measures of body fatness. However, the race-related differences in serum leptin may be due to incomplete adjustment for differences in body fat distribution. Blacks have a higher amount of subcutaneous fat than do whites and secretion of leptin is greater from subcutaneous fat compared to visceral fat. When waist circumference was taken into consideration, the association between race and leptin was no longer present [40].

In this study, no association was found between smoking and serum leptin in the multivariate-adjusted analysis. Previous studies on the association between circulating leptin and lifestyle factors yielded equivocal results [3,6,40,41]. In support of our findings, De Silva et al [41] found no relation between circulating leptin and smoking. In contrast, Donahue et al [6] reported that sex-age-race-ethnicity adjusted leptin concentrations were significantly higher in nonsmokers (14.3 ng/ml) than in smokers (8.4 ng/ml) who smoked more than a pack of cigarettes/d (P < 0.001). It has been postulated that smoking may increase tissue sensitivity to leptin and subsequently lead to lower body weight among smokers [3] which leads to lower concentrations of leptin. Another possible explanation is that smokers tend to have less body fat mass compared to nonsmokers [42] which in turn leads to lower circulating leptin concentrations. Because we adjusted the analysis for BMI, the lack of association between serum leptin and smoking is rather expected.

Serum leptin concentration was independently and directly related to BMI. A direct relation between serum leptin and body composition is in agreement with the observations made by several investigators [1,43]. In a study by Racettet et al [43], leptin concentrations are highly correlated with adiposity measures including BMI, body fat mass, and percentage of body fat. Also, a strong positive correlation (r = 0.85, P < 0.001) was found between serum leptin and the percentage of body fat [1]. Since leptin production was directly related to fat mass, obese subjects had higher serum leptin concentrations when compared to normal-weight subjects (31.3 ng/mL vs. 7.5 ng/mL; P < 0.0001). On the other hand, leptin decreased in response to diet-induced or exercised-induced weight loss [44]. Thus, body fat is an important predictor of serum leptin concentrations. Given a direct relation between BMI and serum triacylglycerol [45], a positive association between serum leptin and serum triacylgycerol observed in this study is along the expected lines.

FFQ is an important dietary assessment tool used in dietary epidemiological studies because the food intake data derived from it is sufficiently valid [46], and it is a practical and economical method [47] with low respondent and investigator burden [48]. Noethlings et al [49] reported that data on portion sizes add little to the variance in food intake, and the major part of variance in food intake is explained by the frequency of food consumption alone. However, cross-sectional studies that use the FFQ tool are prone to measurement error due to the participants' inability to recall dietary intakes precisely. In the NHANES III, dietary data collected using FFQ reflect intakes a month prior to the survey date. It is not known whether this data represent habitual intake of participants. However, dietary patterns derived in this study are in congruence with the commonly reported food intake patterns in the US, i.e., Western and Prudent [16,17,19,20] which in itself lends sufficient validity to the food intake data collected through the use of FFQ in the NHANES III.

Derivation of dietary patterns using the factor analysis is a reproducible and a valid method [31]. Hu et al [20] showed reasonable reproducibility and validity of major dietary patterns defined by factor analysis using food consumption data collected through FFQ. Later, Newby et al [50] in a confirmatory analysis showed a good reproducibility and generalizability of dietary patterns across populations. Dietary patterns derived from the factor analysis were generally stable over a period of time despite changes in eating behavior, food supply, and perceptions of what is regarded as healthy [50]. A limitation of factor analysis was subjectivity used in grouping of individual foods into food groups, extracting number of factors (patterns), and naming dietary patterns [18]. Due to the cross-sectional design of this study, cause and effect analysis is not appropriate.

## Conclusion

In the NHANES III subset population, we found no association between serum leptin concentration and dietary patterns after adjusting for certain covariates suggesting that circulating leptin concentrations are independent of dietary intake patterns. Sex, age, BMI, serum triacyglycerol, and serum insulin are independent predictors of serum leptin concentrations. Further studies are needed to evaluate the effect of diet composition relative to leptin expression to better understand the complicated relationship between diet and leptin.

## Competing interests

The authors declare that they have no competing interests.

## Authors' contributions

EM and VG were responsible for study design and drafting the manuscript. MK was responsible for study design, data acquisition, data management, and data analysis. EM, VG, and MK were responsible for interpretation of results. VG and MK were responsible for revision of the manuscript. At the time of the study, EM was a graduate student and VG was a faculty member in the Departments of Clinical Nutrition and Food and Nutrition, Rush University Medical Center, Chicago. Part of the data comes from the masters thesis of EM and she acknowledges her thesis committee.
